# The Amalgam Question

**Published:** 1861-08

**Authors:** 


### THE AMALGAM QUESTION.

All are familiar with the fact that amalgam plugs in the teeth are frequently blackened, but many seem never to inquire why. They are content to ascribe the blackness to oxydation. That the discoloration is superficial affords them real gratification.

Bearing in mind that *mercury* is a constituent of all *amalgams*, it may be profitable to inquire a little into the cause or causes of the discoloration referred to, and to notice some of the probable consequences of it.

Mercury is ordinarily a white metal, and we are aware of no process by which its color can be very materially changed. In this respect it differs greatly from gold, which can be made to assume any hue, from its own "golden yellow" to a jet black, notwithstanding the statement that "pure gold possesses the same color the world over." It is evident, then, that the discoloration of an amalgam plug is not due to a change in the mechanical arrangement of the particles of the mercury. We are, therefore, to conclude that the blackness results from the combination of some other element, or elements, with the mercury, or that the mercury has nothing to do with the discoloration.

It should be borne in mind that the most popular dental amalgam contains tin and silver, both, as well as mercury, characterized by strong affinities for some of the non-metallic elements liable to be brought in contact with them. The inquiry must, therefore, have reference to these, as well as to mercury.

Oxygen, sulphur, and chlorine are the elements most important to be considered in this connection, with reference to their affinities for these metals.

The question is, what chemical compound, or compounds cause the blackness? If a single compound, it is evidently insoluble; for however black, if soluble, it could only blacken the saliva, not the plug.

Is the black substance a chloride, then? Let us consider. It can not be chloride of tin; for both of its chlorides are soluble. Nor can it be chloride of silver; for, though insoluble, it is white, or grayish. And it is not chloride of mercury, for it is soluble; nor the sub-chloride, for it is white.

Is it an oxyd? Well, not an oxyd of tin, certainly, as the color determines; but both the oxyds of silver, and the sub-oxyd of mercury, are black.

Is it a sulphuret? The sulphuret of silver, and the proto-sulphurets of tin and mercury are all black, or nearly so. We may conclude, then, to discard chlorine, as an agent in the blackening process.

Both sulphur and oxygen are capable of acting with any or all three of the metals under consideration, when the circumstances are favorable. Warmth, moisture, completeness of contact, concentration, or rather, condensation of the elements, and their nascent condition, may be regarded as some of the circumstances likely to promote the action of these elements on the metals under consideration.

The first three of these conditions are always present in the mouth, the liquidity of the saliva bringing itself and the substances dissolved in it into the most intimate contact with any solids retained in the mouth. The fourth condition is afforded with respect to oxygen, as the saliva absorbs, and thereby concentrates this element. And as to the last named modifying circumstance mentioned, that is, the nascent state, the action of both oxygen and sulphur are modified by it in many cases. Hydro-sulphuric acid (sulphureted hydrogen) is very frequently, if not usually present in the mouth. It is often exhaled by the breath, and, being soluble in water, it, of course, is dissolved, to some extent, in the saliva. It is readily decomposed, especially when in contact with substances having an affinity for either one of its elements. As it is decomposed, its sulphur, with all the energy incident to the nascent state, unites with the metals under consideration. And this action is modified by the relative strength of its affinity for the three metals, and by the ratio of their equivalents. If the three affinities were equal, the sulphur would unite with the metals in the proportions of tin, 58, mercury, 101, and silver, 108. But mercury and silver have a stronger affinity for sulphur than tin has ; and, therefore, the force of the sulphur is mainly spent on the former two metals. We would conclude, then, that much of the blackness is due to the formation of sulphurets of silver and mercury.

But all this time the oxygen, concentrated in the saliva, is not idle. In regard to its affinities, it will be sufficiently accurate, for present purposes, to apply the remarks already made in regard to sulphur. Its energies, too, are mainly spent on the silver and mercury. The sub-oxyds of these metals are more likely to be formed, under the circumstances, than the protoxyds; and as these are black, dark colored amalgam plugs are not mysterious.

It may be well to remark, in this connection, that careful analysis fully confirms the conclusions here arrived at by a process of reasoning, and it might have been sufficient, for some, to have stated this at the outset; but we preferred the present course, as better calculated to impress the minds of our younger brethren, whom we regard as the hope of our profession.

Much has been said about galvanic action, in the various discussions of the amalgam question ; and it is plain that the subject is but little understood by many who talk about it. The dull boy in the grammar school, when puzzled about the classification of a word, contents himself with calling it an adverb ; and, in like manner, too many of us, when at a loss in regard to the nature of certain chemical actions, refer them to galvanic influence, and rest content. We have heard of galvanic currents vibrating back and forth, along a gold clasp, till, by their rapid and continued seesawing, they had worn, or cut away the tooth. We have listened to much that was but little farther removed from nonsense. Still the question of galvanic action has its place, and is entitled to consideration. Indeed, it is probable that chemical affinity is only another name for galvanic, or electric influence. Certain it is, however, that when two metals, in contact with each other, are placed in a liquid which acts on but one of them, or unequally on both, a galvanic current is established. The force of this current is modified by the degree of energy of the chemical action, by the extent of surface acted on, or excited, and by the distance of the excited surfaces from each other. There are other modifying circumstances not important to be noticed here.

Bearing these points in mind, let us apply them to amalgam plugs in the mouth.

We have seen that the constituents of the saliva act with unequal energy on the metals considered; and, as they are in the saliva, and in contact, it follows that galvanic action must ensue. And if there is such action at all, in this case, it must result in the formation of an almost infinite series of minute circles, similar to those formed by immersing commercial zinc in a dilute acid. And as the decomposing power of galvanic circles varies inversely as the square root of the distance between the excited surfaces, actual or apparent contact gives a battery of great power. And it should be borne in mind that the exciting solution of a galvanic battery is itself decomposed, or rather, some binary compound in it must undergo decomposition. The saliva, then, acting on one of these metals, or unequally on more than one of them, galvanic action is established, and this being so, it follows that some binary compound of the saliva is decomposed. And if the currents have sufficient force, all its binary compounds will be thus decomposed. Water, hydrosulphuric acid, and the soluble chlorides would thus readily undergo decomposition. It follows from this, that the formation of the oxyds and sulphurets, referred to above, is promoted by the galvanic action thus established.

But even to this there is a limit. The oxyds and sulphurets thus formed, being nearly insoluble in ordinary saliva, and being deposited on the surfaces of the metals, protect the latter from the action of the former, and thus by arresting, to a good degree, the chemical, arrest, with it, the galvanic action. This will be better understood by a brief consideration of the ordinary zinc and copper battery. A sheet of zinc and one of copper, connected by a wire, if plunged into water, immediately establish a galvanic current. But this current is soon arrested ; for the oxyd of zinc, formed on the surface of the zinc plate, is insoluble in the water, and, therefore, the metal is protected from farther action. But if a little sulphuric acid be added to the water, it dissolves the oxyd, as fast as formed, and the zinc continues to decompose the water, and the galvanic current is kept up.

It is only on account of this insolubility of the black oxyd and sulphuret of mercury, thus formed, that we see so few cases of the constitutional effects of the metal, resulting from amalgam fillings ; for if they were as soluble as the chloride, a single plug of ordinary size could scarcely fail to produce these effects. If this position be correct, it follows that if it is proper at all to use amalgam fillings, it is no cause for discouragement to see them turn black. On the other hand, it is rather fortunate that they do blacken. And this corresponds exactly with our own observation on the subject, which has been *very extensive*, and embraces a period of more than a dozen years. We do not now recollect a single case of constitutional disturbance resulting from the presence of amalgam fillings, in which the blackening of the plugs was very decided ; and in the last three cases that we observed, there was none at all.

Genuine chemical combination takes place in the formation of amalgams. This is evidenced by various phenomena that may be observed. When an attempt is made to combine two metals, differing considerably in their affinities for non-metallic elements, one of them, as soon as contact is made, necessarily becomes positive, and the other negative. For example, in making an amalgam of gold, the mercury is rendered positive, and its affinity for oxygen, a highly electro-negative element, is thereby greatly increased. The consequence is that the black oxyd of mercury is abundantly formed. The same thing takes place, though to a less extent, in making amalgams of tin and silver. We have met with many who regard the presence of this black oxyd as an evidence that the mercury is not pure. It is formed, however, when the metals are all pure. If this oxyd is put into a tooth cavity, along with the amalgam, of course the plug will not make as good an appearance as when it is removed by washing with alcohol, water, or a solution of common salt. And when the oxyd is thus washed away, there is less danger of constitutional disturbance than when it is allowed to remain ; for, in most mouths, after the amalgam is inserted, the sulphuret of mercury will be formed in greater abundance than the oxyd, and it is well known that the latter is a more active poison than the former. And besides its greater activity, in contact with hydrochloric acid, which is presented in many mouths, it is readily decomposed, the reaction yielding water and the subchloride of mercury, or calomel. It is well known that calomel is a still more active preparation of mercury than the sub-oxyd.

But, as the fluids of the mouth can not act unequally on the metals under consideration without exciting galvanic action, it will be understood by all who remember the presence of soluble chlorides in the saliva, that the formation of the subchloride, or even the chloride of mercury, is not improbable. The chlorides of sodium, and potassium, being binary compounds, and soluble, are readily decomposed by galvanic action. The chlorine, thus liberated, will unite with the mercury, just as the liberated oxygen unites with the zinc of the ordinary battery. As these chlorides are present in normal saliva, there is nothing remarkable in the formation of chloride of mercury from amalgam plugs ; and it is probable that in all cases of constitutional effects arising from their presence, either the subchloride or the chloride is the agent that produces them. Were it not that the breaths of most patients, as well as their saliva, are loaded with hydrosulphuric acid, so that the insoluble black sulphuret is formed, the formation of at least one of the chlorides of mercury could scarcely be prevented.

We may continue this subject. W.

